# ﻿Diversity and distribution of the Isopoda (Crustacea, Peracarida) of Kuwait, with an updated checklist

**DOI:** 10.3897/zookeys.1080.71370

**Published:** 2022-01-05

**Authors:** Manal Abdulrahman Al-Kandari, Valiallah Khalaji-Pirbalouty, Hadeel Abdulkhaliq, Weizhong Chen

**Affiliations:** 1 Ecosystem-Based Management of Marine Resources, Environment, and Life Sciences Research Center, Kuwait Institute for Scientific Research, Hamad Al-Mubarak Street, Building 900004, Area 1, Raas Salmiya, Kuwait Kuwait Institute for Scientific Research Hawally Kuwait; 2 Department of Biology, Faculty of Basic Science, Shahrekord University, Shahrekord, Iran Shahrekord University Shahrekord Iran

**Keywords:** Biodiversity, checklist, first records, geographical distribution, Isopoda, Kuwait

## Abstract

Thirty-eight species of Isopoda, belonging to 13 families and 29 genera, are listed from Kuwait based on previous literature records (of 17 species) and collections carried out along Kuwait’s coastal and subtidal zones during the present study. The majority of species belongs to the suborder Cymothoida (23), followed by Sphaeromatidea (9), Oniscidea (3), Valvifera (2), and Asellota (1). In total, 25 species were collected and identified from 12 families and 22 genera from Kuwaiti coastal and subtidal areas. These include eight families, 15 genera, and 21 species recorded for the first time from Kuwait. Isopod diversity was highest in the sandy rock areas, including southern Kuwait, particularly in Al-Khiran and Al-Nuwaiseeb, and in mixed habitat (muddy, rocky, and sandy) intertidal transects such as in Failaka Island. The species number increased from the subtidal and lowest zones into the high tidal zone. Isopods were found in sandy substrata, among shells, cobbles, rocks, dead corals, and algae.

## ﻿Introduction

The isopod fauna in Kuwait’s intertidal and subtidal habitats have received little attention. The few significant accounts of Kuwait’s marine isopods are those of Bowman and Tareen (1983), describing six new species of Cymothoidae. In addition, Abu-Hakima (1984) recorded a bopyrid, *Epipenaeonelegans* Chopra, 1923, and Jones (1986) in ‘Field Guide to the Seashores of Kuwait’ recorded six marine isopods from Kuwait. However, *Apanthurasandalensis* Stebbing, 1900, *Ligiaexotica* Roux, 1828; and *Cymodocerichardsoniae* Nobili, 1906, were misidentified in his guide. They are reidentified as *Amakusanthura* sp., *L.persica* Khalaji-Pirbalouty & Wägele, 2010, and *C.delvarii* Khalaji-Pirbalouty, Bruce & Wägele, 2013, respectively, in this work.

*Arcturinoidesangulata* Kensley, Schotte & Poore, 2007 and *Astacillamccaini* Kensley, Schotte & Poore, 2007 have been reported by Kensley et al. (2007) from the coasts of Kuwait and Saudi Arabia and, most recently, Jones and Nithyanandan (2012) reported four species of the genus *Eurydice* Leach, 1815 from Kuwait and Saudi Arabia. In contrast, the isopod fauna along the Iranian coast of the Gulf has received more attention than adjacent regions (e.g., Khalaji-Pirbalouty and Wägele 2009, 2010a, b, c, 2011, 2012; Khalaji-Pirbalouty et al. 2013; Khalaji-Pirbalouty and Bruce 2014; Khalaji-Pirbalouty and Raupach 2014, 2016).

In 2013, a large-scale survey covering Kuwait’s entire coastline and offshore islands was initiated to document biodiversity, species distribution, and species abundance of the intertidal fauna. This survey was completed in 2017 (Al-Kandari et al. 2017). A further complementary sampling of four sites was conducted from 2016 to 2018. Survey results for molluscs, decapods, and polychaetes have been published (Al-Kandari et al. 2019a, b, 2020a, b), and summaries on other taxa are in progress. Here we report the results for the crustacean order Isopoda.

## ﻿Materials and methods

### ﻿Intertidal and subtidal sampling

**Figure 1.**
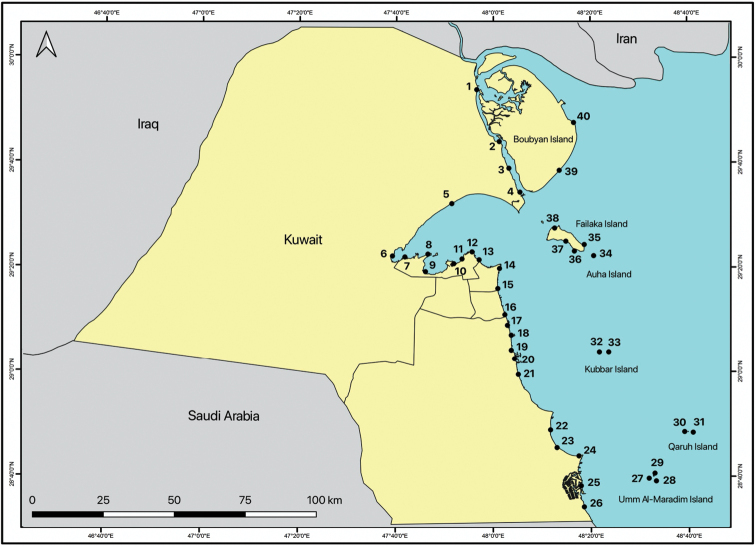
Map of the 40 sampling sites established in Kuwait’s intertidal and subtidal zones; site numbers corresponding to Table 1.

Thirty-eight intertidal transects and two subtidal sites were sampled quantitatively and qualitatively for macrofauna (Fig. 1, Table 1). Transects were located between Khor Al-Subiya in the north and the border with Saudi Arabia in the south. The surveys were conducted in daylight during the late autumn and winter seasons from December 2013 to December 2016. The sampling dates (see Table 1) and time for each site coincided with the lowest tides (as near to 0 chart datum as possible) using the Kuwait Port Authority’s Tide Tables for 2013, 2014, 2015, and 2016. Kuwait’s intertidal areas consist of coral, rocky, sandy, and/or muddy habitats or combinations thereof. At some transects, sandy mud or muddy sand covered a hard stratum throughout the intertidal range. Other transects consisted of combinations of sand and rocks, with some rocks lose and resting on the surface and others being part of the bedrock. All sandy beaches, rocky beaches, underneath stones, rubble, algal turf, and/or seagrass beds were sampled at each transect. Samples were left in seawater for a day before the fauna was collected from the bottom of the containers. Additionally, fauna living within porous rocks was collected by breaking the rocks with a hammer, placing the resulting debris in isotonic magnesium chloride solution, and collecting the fauna after their relaxation. For soft substrates, a 25 × 25-cm square metal box corer, 15 cm deep, was placed randomly, and sediment was collected by spade from inside the corer. These samples were sieved with seawater using 0.3-mm mesh sieves 45- and 75-cm in diameter, and all sediment and organisms remaining were preserved with 5% buffered formalin for subsequent picking and identification. Isopod specimens were also collected qualitatively from under rocks and among intertidal vegetation. Sand was sieved further samples were collected from rocks broken by a hammer, washing algae, sponges and seagrass, turning over stones, as well as collecting directly in the habitat. Material was rinsed under seawater, and all the washings passed through a 0.3-mm mesh sieve to collect any isopod specimens. The collected isopods were fixed in a 75–95% ethanol solution for subsequent morphological and molecular analyses. All specimens were deposited in the Kuwait Institute for Scientific Research (**KISR**) reference collection.

**Table 1.** Sampling sites studied in the intertidal and subtidal zones of Kuwait with habitat details (*KPC = Kuwait Petroleum Corporation).

Site No.	Site Name (north to south)	Position			
		Sampling Dates	Coordinates	Area	Substrate
**Khor Al-Subiya: north and west of Boubyan Island (BI)**					
1	Umm Al-Shajar (north Khor Al-Subiya), (BI1)	29.12.2015	29°54.263'N, 48°01.475'E	BI	Mud
2	Khor Al-Subiya (Al-Magasel)	23.11.2014	29°44.476'N, 48°05.740'E	North	Mud-rock
3	Khor Al-Subiya (Al-Shumaima)	24.11.2014	29°39.403'N, 48°07.850'E	North	Mud-rock
4	Khor Subiyah (south)	25.11.2014	29°34.849'N, 48°10.248'E	North	Mud
**Kuwait Bay**					
5	Mudairah	30.12.2014	29°32.672'N, 47°55.394'E	Bay-mud	Mud
6	Al-Kuwaisat	17.11.2014	29°22.677'N, 47°42.480'E	Bay-mud	Mud
7	Al-Judailiat	02.02.2014	29°22.497'N, 47°45.183'E	Bay	Sand-rock
8	Aushairij	03.02.2014	29°23.047'N, 47°50.192'E	Bay	Sand-rock
9	Sulaibikhat Bay	06.11.2014	29°19.702'N, 47°49.670'E	Bay	Mud
10	Shuwaikh (KPC*), subtidal	22.02.2016	29°21.401'N, 47°56.390'E	Bay	Sand-rock
11	Kuwait Bay (Al-Salam Beach)	09.12.2013	29°21.631'N, 47°57.204'E	Bay	Sand-rock
12	Kuwait Bay (Ras Ajuza)	08.12.2013	29°23.481'N, 47°59.800'E	Bay	Sand-rock
**East Kuwait Bay**					
13	Al-Sha'Eab	19.01.2014	29°21.979'N, 48°01.344'E	Middle 1	Sand-rock
14	Al-Salmiya	19.12.2013	29°20.313'N, 48°05.775'E	Middle 1	Sand-rock
**South Kuwait Bay**					
15	Al-Messilah	18.12.2013	29°16.496'N, 48°05.407'E	Middle 1	Sand-rock
16	Al-Funaitees	19.12.2013	29°11.519'N, 48°06.938'E	Middle 1	Sand-rock
17	Abu Halifa	04.01.2014	29°08.154'N, 48°07.985'E	Middle 2	Sand-rock
18	Al-Mangaf	01.02.2014	29°06.041'N, 48°08.323'E	Middle 2	Sand-rock
19	Masfat Al-Ahmadi	10.12.2014	29°04.431'N, 48°08.676'E	Middle 2	Sand-rock
20	North Oil loading terminal, subtidal	28.09.2014	29°8.043'N, 48°09.139'E	Middle 2	Sand-rock
21	Mina Abdullah	16.02.2014	29°00.071'N, 48°09.853'E	Middle 2	Sand-rock
22	Al-Julaia'Ea	17.02.2014	28°49.480'N, 48°16.812'E	South	Sand-rock
23	Dohat Al-Zour	02.03.2014	28°46.100'N, 48°18.210'E	South	Sand-rock
24	Ras Al-Zour	08.01.2015	28°44.502'N, 48°22.950'E	South	Sand-rock
25	Al-Khiran	03.03.2014	28°38.813'N, 48°23.429'E	South	Sand-rock
26	Al-Nuwaiseeb	04.03.2014	28°34.794'N, 48°24.078'E	South	Sand-rock
**Islands**					
27	Umm Al-Maradim Island, east (UI1)	11.11.2014	28°40.778'N, 48°39.207'E	UI1	Sand-rock
28	Umm Al-Maradim Island, northeast (UI2)	11.11.2014	28°40.939'N, 48°39.196'E	UI2	Sand-rock
29	Umm Al-Maradim Island, northwest	11.11.2014	28°40.960'N, 48°39.173'E	UI3	Sand-rock
30	Qaruh Island (north), (QI1)	10.11.2014	28°49.105'N, 48°46.553'E	QI	Sand-rock
31	Qaruh Island (south), (Q2)	10.11.2014	28°49.022'N, 48°46.607'E	QI	Sand-rock
32	Kubbar Island (east), (Q3)	09.11.2014	29°04.278'N, 48°29.655'E	KI	Sand-rock
33	Kubbar Island (west)	09.11.2014	29°04.377'N, 48°29.472'E	KI	Sand-rock
34	Auha Island (northwest), (AI)	10.02.2016	29°22.726'N, 48°26.269'E	AI	Sand-rock
35	Failaka Island (east 2), (FI1)	25.12.2014	29°23.710'N, 48°24.136'E	FI	Sand-rock
36	Failaka Island (east 1), (F2)	24.12.2014	29°23.629'N, 48°23.958'E	FI	Sand-rock
37	Failaka Island (south), (FI3)	23.12.2014	29°25.625'N, 48°20.307'E	FI	Mud-rock
38	Failaka Island (northwest), (FI4)	22.12.2014	29°28.049'N, 48°17.838'E	FI	Mud-rock
39	Boubyan Island (south), (BI2)	24.01.2015	29°38.993'N, 48°18.830'E	BI	Mud
40	Boubyan Island (Ras Al-Gayed), (BI3)	25.01.2015	29°48.093'N, 48°21.975'E	BI	Mud

### ﻿Species identification

For identification, morphological studies were conducted using a Leica DFC450 camera mounted on a Leica M125 Stereomicroscope equipped with an imaging system that was employed to obtain colour images of the specimens. For greater depth of field, we merged 10–20 source images of a single specimen taken at different focus distances into one final image with the software LAS V4.5. The final image was edited using Adobe Photoshop. Isopods were identified to the lowest possible taxonomic level.

## ﻿Results

In total, 25 species representing 12 families and 22 genera were identified from specimens collected in the present study. These species were collected from 31 intertidal transects, including 17 mainland and 14 island transects, and two subtidal sites (Table 2).

**Table 2.** List of isopod species recorded in Kuwait in the present survey (* indicates a new record to Kuwait) and from literature records.

Suborder	Family	Species	Reference
CYMOTHOIDA	Anthuridae	*Amakusanthura* sp. *	This study
CYMOTHOIDA	Expanathuridae	*Eisothistos* sp. *	This study
CYMOTHOIDA	Cirolanidae	Atarbolanaexoconta*	This study
CYMOTHOIDA	Cirolanidae	Baharilanakiabii*	This study
CYMOTHOIDA	Cirolanidae	Cirolanatarahomii*	This study
CYMOTHOIDA	Cirolanidae	*Eurydicearabica*	Jones and Nithyanandan (2012)
CYMOTHOIDA	Cirolanidae	*E.marzouqui*	Jones & Nithyanandan, 2012
CYMOTHOIDA	Cirolanidae	*E.peraticis*	Jones and Nithyanandan (2012); This study
CYMOTHOIDA	Cirolanidae	*Metacirolana* sp. *	This study
CYMOTHOIDA	Corallanidae	Lanociragardineri*	This study
CYMOTHOIDA	Cymothoidea	*Anilocramonoma*	Bowman & Tareen, 1983
CYMOTHOIDA	Cymothoidea	*Catoessagruneri*	Bowman & Tareen, 1983
CYMOTHOIDA	Cymothoidea	*Cymothoaeremita*	Bowman & Tareen, 1983
CYMOTHOIDA	Cymothoidea	*Jorymasawayah*	Bowman & Tareen, 1983
CYMOTHOIDA	Cymothoidea	*Mothocya* sp.	Bowman & Tareen, 1983
CYMOTHOIDA	Cymothoidea	*Nerocilaarres*	Bowman & Tareen, 1983
CYMOTHOIDA	Cymothoidea	*N.kisra*	Bowman & Tareen, 1983
CYMOTHOIDA	Cymothoidea	*N.sigani*	Bowman & Tareen, 1983
CYMOTHOIDA	Cymothoidea	*N.phaiopleura*	Bowman & Tareen, 1983
CYMOTHOIDA	Gnathiidae	*Gnathia* sp.*	This study
CYMOTHOIDA	Gnathiidae	*Elaphognathia* sp.*	This study
CYMOTHOIDA	Bopyridae	*Epipenaeonelegans*	Abu-Hakima, 1984
CYMOTHOIDA	Bopyridae	*Parabopyrella* sp.*	This study
ONISCIDE	Ligiidae	Ligiapersica*	This study
ONISCIDE	Olibrinidae	Olibrinusantennatus*	This study
ONISCIDE	Tylidae	*Tylosmaindroni*	Taiti and Ferrara(1991); this study
SPHAEROMATIDEA	Sphaeromatidae	Cymodocedelvarii*	This study
SPHAEROMATIDEA	Sphaeromatidae	C.fuscina*	This study
SPHAEROMATIDEA	Sphaeromatidae	C.waegelei*	This study
SPHAEROMATIDEA	Sphaeromatidae	Dynamenellagranulata*	This study
SPHAEROMATIDEA	Sphaeromatidae	Heterodinamccaini*	This study
SPHAEROMATIDEA	Sphaeromatidae	Sphaeromakhalijfarsi*	This study
SPHAEROMATIDEA	Sphaeromatidae	S.walkeri*	This study
SPHAEROMATIDEA	Sphaeromatidae	*S.annandalaei*	This study
SPHAEROMATIDEA	Sphaeromatidae	Sphaeromopsissarii*	This study
VALVIFERA	Arcturidae	*Arcturinoidesangulata*	Kensley et al. (2007); this study
VALVIFERA	Arcturidae	*Astacillamccaini*	Kensley et al. (2007); this study
ASELLOTA	Paramunnidae	*Heterosignum* sp.*	This study

Sphaeromatidae Latreille, 1825 was the best-represented family with five genera and eight species, followed by the family Cirolanidae comprising five genera and five species. Two species were recorded in each of the families Gnathiidae and Arcturidae. The remaining seven families were represented by single species (Table 2). In descending order, the most widely distributed isopod species were *Amakusanthura* sp. from 20 transects, *Gnathia* sp., and *Sphaeromopsissarii* Khalaji-Pirbalouty & Wägele, 2009, from 18 transects, *Astacillamccaini* Kensley, Schotte & Poore, 2007, from 15 transects; *Heterodinamccaini* Schotte & Kensley, 2005, from 12 transects; *Cymodocedelvarii* Khalaji-Pirbalouty, Bruce & Wägele, 2013, occurred at 12 transects, and *Lanociragardineri* Stebbing, 1904, was collected from ten transects. Interestingly, some species occurred in their 100s from single qualitative samples. Such high numbers for *S.sarii* and *C.delvarii* were obtained from randomly collected *Sargassum* at Al-Nuwaiseeb and Failaka Island. Similarly, high numbers of *S.sarii* occurred on algal turfs from Kubbar Island. Other species found in high numbers were found from rocks, dead coral, or dead shells and included *Gnathia* sp., *H.mccaini*, and *Sphaeromawalkeri* Stebbing, 1905.

Thirty-eight isopod species under five sub-orders, 13 families, and 29 genera are listed in taxonomic order, including Kuwait’s previous records (17 species), type localities, and geographical distributions.

## ﻿Taxonomy

**Order Isopoda Latreille, 1817**

### ﻿Suborder Cymothoida Wägele, 1989

**Superfamily Anthuroidea Leach, 1814**

#### Family Anthuridae Leach, 1814

##### Genus *Amakusanthura* Nunomura, 1977

###### ﻿ Amakusanthura

Taxon classificationAnimaliaIsopodaAnthuridae

sp.

BD136C53-97FA-5215-89AD-A64A56933741

Figure 2A

Apanthura sandalensis ­— Jones, 1986: 148, pl. 40 [not Apanthurasandalensis Stebbing, 1900; misidentification].

####### Material examined.

Kuwait. 3 specimens; St. 2; 29°44.476'N, 48°05.740'E; 23 Nov. 2014; ♀♀, 2 juveniles; St. 3; 29°39.403'N, 48°07.850'E; 24 Nov. 2014; (5 ♀♀); St. 4; 29°34.849’ N, 48°10.248'E; 25 Nov. 2014, 2 ♂♂; St. 8; 29°23.047'N, 47°50.192'E; 3 Feb. 2014; 1 ♀; St. 11; 29°21.631'N, 47°57.204'E; 9 Dec. 2013; 2 ♀♀; St. 12; 29°23.481'N, 47°59.800'E; 8 Dec. 21013; (1 ♀), 1 juvenile; St. 18; 29°06.041'N, 48°08.323'E; 1 Feb. 2014; 3 ♀♀; St. 19; 29°04.431'N, 48°08.676'E; 10 Dec. 2014; 2 ♀♀; St. 22; 28°49.480'N, 48°16.812'E; 17 Feb 2014, 1 juvenile, 6 ♀♀; St. 24; 28°44.502'N, 48°22.950'E; 08 Jan. 2015; 1 ♀; St. 25; 28°38.813'N, 48°23.429'E; 3 Mar. 2014; 5 ♀♀, 2 juveniles; St. 26; 28°34.794'N, 48°24.078'E; 4 Mar. 2014; 5 ♀♀, 3 juveniles; St. 27; 28°40.778'N, 48°39.207'E;; 11 Nov. 2014; 3 ♀♀; St. 28; 28°40.939'N, 48°39.196'E; 11 Nov. 2014; 1 ♀; St. 29; 28°40.960'N, 48°39.173'E; 11 Nov. 2014; 2 ♀♀; St. 30; 28°49.105'N, 48°46.553'E; 10 Nov. 2014; 7 ♀♀; St. 32; 29°04.278'N, 48°29.655'E; 9 Nov. 2014; 12 ♀♀; St. 34; 29°22.726'N, 48°26.269'E; 10 Feb. 2014; 3 ♀♀; St. 36; 29°23.629'N, 48°23.958'E; 24 Dec. 2014; 2 ♀♀; St. 38; 29°28.049'N, 48°17.838'E; 22 Dec. 2014.

####### Remarks.

*Amakusanthuramotasi* (Negoescu, 1980) is the only species of this genus recorded from the nearest locality (Gulf of Aden). The specimens examined here differ from *A.motasi* in the shape of the pleon with different lengths of pleonites 1–5 (vs. pleonites 1–5 similar to each other in *A.motasi*), the setation of pereopods, uropods and pleotelson; antenna and antennular articles are narrower than in *A.motasi*.

**Figure 2.**
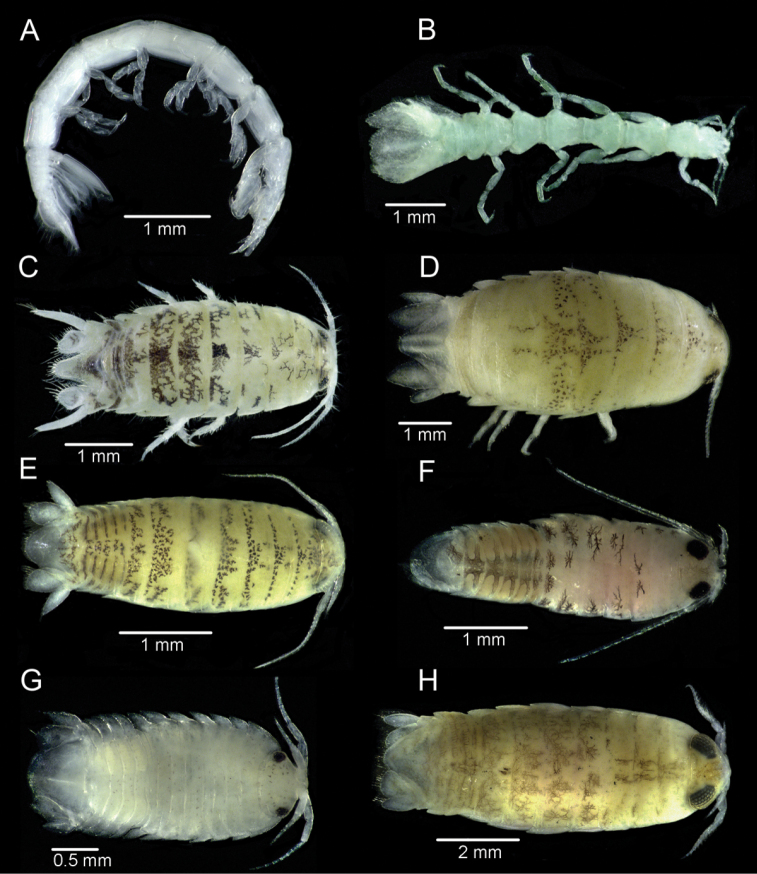
**A***Amakusanthura* sp. from Kubbar Island **B***Eisothistos* sp. from Failaka Island **C***Atarbolanaexoconta* Bruce & Javed, 1987 from Masfat Al-Ahmadi **D***Baharilanakiabii* Khalaji- Pirbalouty & Wägele, 2011from Al-Nuwaiseeb **E***Cirolanatarahomii* Khalaji-Pirbalouty & Wägele, 2011 from Quaruh Island **F***Eurydiceperaticis* Jones, 1974 from Alkhiran **G***Metacirolana* sp. from Um-Almaradim **H***Lanociragardineri* Stebbing, 1904 from Al-Shamaimah.

####### Distribution.

New record for Kuwait.

#### Family Expanathuridae Poore, 2001

##### Genus *Eisothistos* Haswell, 1884

###### ﻿ Eisothistos

Taxon classificationAnimaliaIsopodaExpanathuridae

sp.

7CD2EB0A-C6D9-5CE4-9198-A38DC4429B7E

Figure 2B

####### Material examined.

1 ♂; St. 38; 29°28.049'N, 48°17.838'E; 22 Dec. 2014.

####### Distribution.

New record for Kuwait

#### Family Cirolanidae Dana, 1852

##### Genus *Atarbolana* Bruce & Javed, 1987

###### ﻿ Atarbolana exoconta

Taxon classificationAnimaliaIsopodaExpanathuridae

Bruce & Javed, 1987

E94991AF-292B-5446-9AF9-F11CBBB65E01

Figure 2C

Atarbolana exoconta Bruce & Javed, 1987: 145, figs 1, 2, Manora Island, Pakistan (type locality); Khalaji-Pirbalouty & Raupach, 2016: 155–162, figs 2–6.

####### Material examined.

4 ♂♂, 5 ♀♀; St. 19; 29°04.431'N, 48°08.676'E; 10 Dec. 2014; 1 ♂, 8 ♀♀; St. 21; 29°00.071'N, 48°09.853'E; 16 Feb. 2014; 2 ♀♀: St. 27; 28°40.778'N, 48°39.207'E; 11 Nov. 2014.

####### Distribution.

Pakistan, Oman Sea (Bruce and Javed 1987; Khalaji-Pirbalouty and Raupach 2016), new record for Kuwait.

##### Genus *Baharilana* Bruce & Svavarsson, 2003

###### ﻿ Baharilana kiabii

Taxon classificationAnimaliaIsopodaExpanathuridae

Khalaji-Pirbalouty & Wägele, 2011

1C5A4C11-46D3-5467-8122-CDFF9EAAD063

Figure 2D

Baharilana kiabii Khalaji-Pirbalouty & Wägele, 2011: 34–39, figs 1–4; Qeshm Island, Iran (type locality).

####### Material examined.

1 ♀, 1 juvenile; St. 19; 29°04.431'N, 48°08.676'E; 10 Dec. 2014; 2 ♀♀; St. 25; 28°38.813'N, 48°23.429'E; 3 Mar. 2014; 3 ♂♂, 5 ♀♀; St. 26; 28°34.794'N, 48°24.078'E; 4 Mar. 2014; 1 ♂, 1 juvenile; St. 27; 28°40.778'N, 48°39.207'E; 11 Nov. 2014; 1 ♀, 1 juvenile; St. 32; 29°04.278'N, 48°29.655'E; 9 Nov. 2014; 1 ♀; St. 35; 29°23.710'N, 48°24.136'E; 25 Dec. 2014.

####### Distribution.

Qeshm Island, Hengam Island, Iran (Khalaji-Pirbalouty and Wägele 2011), new record for Kuwait.

##### Genus *Cirolana* Leach, 1818

###### ﻿ Cirolana tarahomii

Taxon classificationAnimaliaIsopodaExpanathuridae

Khalaji-Pirbalouty & Wägele, 2011

DD5AF440-9865-5202-8592-7B94A07E966C

Figure 2E

Cirolana tarahomii Khalaji-Pirbalouty & Wägele, 2011: 39–45, figs 5–8; Qeshm Island, Iran (type locality).

####### Material examined.

7 ♀♀, 3 juveniles; St. 30; 28°49.105'N, 48°46.553'E; 10 Nov. 2014; 1 ♀, St. 32; 29°04.278'N, 48°29.655'E; 9 Nov. 2014.

####### Distribution.

Qeshm Island, Iran (Khalaji-Pirbalouty and Wägele 2011), new record for Kuwait.

##### Genus *Eurydice* Leach, 1815

###### ﻿ Eurydice arabica

Taxon classificationAnimaliaIsopodaExpanathuridae

Jones, 1974

F4282042-FE79-5136-8194-D78A524C055F

Eurydice arabica Jones, 1974: 202, fig. 2, Red Sea (type locality); Bruce, 1986: 221.

####### Distribution.

Kuwait, Al-Ahmad Sea City waterways, Bahrain, Mashtan Island (Jones and Nithyanandan 2012).

###### ﻿ Eurydice marzouqui

Taxon classificationAnimaliaIsopodaExpanathuridae

Jones & Nithyanandan, 2012

8B98574E-A443-5EC8-9C30-9F05FEBC460C

Eurydice marzouqui Jones & Nithyanandan, 2012: 47–48, figs 1–4; Tarut Bay, Saudi Arabia (type locality).

####### Distribution.

Sabah Al-Ahmad Sea City Waterways, Kuwait; Manifa, Saudi Arabia (Jones and Nithyanandan 2012).

###### ﻿ Eurydice peraticis

Taxon classificationAnimaliaIsopodaExpanathuridae

Jones, 1974

6D4CFC03-17B5-54A5-9E94-152340F678DE

Figure 2F

Eurydice peraticis Jones, 1974: 204, fig. 3, Dammam, Saudi Arabia (type locality); Eleftheriou & Jones, 1976: 387; Bruce, 1986: 221; Kazmi et al. 2002: 91, fig. 66.

####### Material examined.

1 ♂; St. 8; 29°23.047'N, 47°50.192'E; 3. Feb. 2014; 2 ♀♀; St. 19; 29°04.431'N, 48°08.676'E; 10 Dec. 2014; 2 ♀♀; St. 21; 29°00.071'N, 48°09.853'E; 16 Feb. 2014; 2 ♂♂, 3 ♀♀; St. 25; 28°38.813'N, 48°23.429'E; 3 Mar. 2014; 1 ♀; St. 34; 29°22.726'N, 48°26.269'E; 10 Feb. 2016; 3 ♂♂, 7 ♀♀; St. 39; 29°38.993'N, 48°18.830'E; 24 Jan. 2015; 6 ♂♂, 9 ♀♀, 2 juveniles; St. 40; 25 Jan. 2015.

####### Distribution.

Saudi Arabia, Bahrain, India, Pakistan, Kuwait (Eleftheriou and Jones 1976; Kazmi et al. 2002).

##### Genus *Metacirolana* Nierstrasz, 1931

###### ﻿ Metacirolana

Taxon classificationAnimaliaIsopodaExpanathuridae

sp.

A7FAE570-155F-5549-B803-D7A0A4AD3BE2

Figure 2G

####### Material examined.

1 ♂; St.2; 29°44.476'N, 48°05.740'E; 23 Nov. 2014; 4 ♀♀; St. 3; 29°39.403'N, 48°07.850'E; 24 Nov. 2014; 1 ♂; St.27; 28°40.778'N, 48°39.207'E; 11Nov. 2014; 1 ♂, 2 ♀♀; St.30; 28°49.105'N, 48°46.553'E; 10 Nov. 2014; 1 ♀; St. 34; 29°22.726'N, 48°26.269'E; 10 Feb. 2016; 2 ♂♂, 2 ♀♀; St. 36; 29°23.629'N, 48°23.958'E; 24 Dec. 2014.

####### Distribution.

New record for Kuwait.

#### Family Corallanidae Hansen, 1890

##### Genus *Lanocira* Hansen, 1890

###### ﻿ Lanocira gardineri

Taxon classificationAnimaliaIsopodaExpanathuridae

Stebbing, 1904

55F0F564-7242-5C0F-B779-859B28D02BF8

Figure 2H

Lanocira gardineri Stebbing, 1904: 706, pl. LI, A, Mahlosmadulu Atoll, Maldive Islands (type locality).
A comprehensive synonymy to the species can be found in Bruce and Sidabalok 2011: 25.

####### Material examined.

1 ♂, 4 ♀♀, 3 juveniles; St. 3; 29°39.403'N, 48°07.850'E; 24 Nov. 2014; 2 ♀♀; St. 12; 29°23.481'N, 47°59.800'E; 8 Dec. 2104; 1 ♀; St. 22; 28°49.480'N, 48°16.812'E; 17 Feb. 2014; 1 Juvenile; St. 30; 28°49.105'N, 48°46.553'E; 10 Nov. 2014; 2 ♂♂, 5 ♀♀; St. 3; 29°39.403'N, 48°07.850'E; 24 Nov. 2014; 3 ♂♂, 6 ♀♀; St. 35; 29°23.710'N, 48°24.136'E; 25 Dec. 2014; 2 ♂♂, 5♀♀, 2 ovigerous ♀♀, 5 juveniles; St. 36; 29°23.629'N, 48°23.958'E; 24 Dec. 2014; 1 ♀; St. 37; 29°25.625'N, 48°20.307'E; 23 Dec. 2014; 1 ♂; St. 38; 29°28.049'N, 48°17.838'E; 22 Dec. 2014; 2 ♂♂; St. 40; 29°48.093'N, 48°21.975'E; 20 Jan. 2015.

####### Distribution.

Maldives, Kenya, Madagascar (Delaney 1989); Western Australia (Bruce and Sidabalok 2011); Iran (Khalaji-Pirbalouty, unpublished), new family for Kuwait.

#### Family Cymothoidae Leach, 1814

Of the cymothoid isopods (parasites of fishes), the following species have been reported by Bowman and Tareen (1983).

##### ﻿ Anilocra monoma

Taxon classificationAnimaliaIsopodaExpanathuridae

Bowman & Tareen, 1983

8367960F-7CF2-54CE-A2DB-79DAE6C2E23F

Anilocra monoma Bowman & Tareen, 1983: 1, figs 3, 4, Kuwait (type locality).

###### Distribution.

Kuwait (Bowman and Tareen 1983).

##### ﻿ Catoessa gruneri

Taxon classificationAnimaliaIsopodaExpanathuridae

Bowman & Tareen, 1983

694C969C-D652-555F-B7DB-C7F13E789E78

Catoessa gruneri Bowman & Tareen, 1983: 18, figs 14, 15, Kuwait (type locality).

###### Distribution.

Kuwait (Bowman and Tareen 1983).

##### ﻿ Joryma sawayah

Taxon classificationAnimaliaIsopodaExpanathuridae

Bowman & Tareen, 1983

FBFB526F-3534-5348-907A-F9CE9AB9B78E

Joryma sawayah Bowman & Tareen, 1983: 21, figs 16–18, Doha, Kuwait (type locality).
Livoneca sp., Mathews & Samuel, 1987: 144.

###### Distribution.

Kuwait (Bowman and Tareen 1983).

##### ﻿ Nerocila arres

Taxon classificationAnimaliaIsopodaExpanathuridae

Bowman & Tareen, 1983

2D168B58-ADBD-52F9-B36E-B291ED09017A

Nerocila arres Bowman & Tareen, 1983: 12, figs 10–12; Kuwait (type locality).
Nerocila kisra Bowman & Tareen, 1983: 8, figs 6–8.

###### Distribution.

Kuwait (Bowman and Tareen 1983).

##### ﻿ Nerocila sigani

Taxon classificationAnimaliaIsopodaExpanathuridae

Bowman & Tareen, 1983

73AEEDC7-A449-58F0-BBDE-15CC5216E0E1

Nerocila sigani Bowman & Tareen, 1983: 12, fig. 9; Kuwait (type locality).

###### Distribution.

Kuwait (Bowman and Tareen 1983).

##### ﻿ Nerocila phaiopleura

Taxon classificationAnimaliaIsopodaExpanathuridae

Bleeker, 1857

620F65E4-F224-50E2-9312-C63DDE1885FC

Nerocila phaiopleura Bleeker, 1857: 25–26, fig. 3, Java (type locality); Bowman & Tareen, 1983: 5, fig. 5.

###### Distribution.

A widespread species, recorded in the Indian Ocean from Hong Kong to South Africa (Bowman and Tareen 1983).

##### ﻿ Mothocya

Taxon classificationAnimaliaIsopodaExpanathuridae

sp.

F08CC190-A0CE-5E01-90B4-69E19C3F68CC

Mothocya sp., Bowman & Tareen, 1983: 25, fig. 19.
Cymothoa eremita ? (Brunnich, 1783), Bowman & Tareen, 1983: 25, fig. 20, India (type locality).

###### Distribution.

India (Bowman and Tareen 1983)

#### Family Gnathiidae Leach, 1814

##### Genus *Gnathia* Leach, 1814

###### ﻿ Gnathia

Taxon classificationAnimaliaIsopodaGnathiidae

sp.

83AAF206-E7E5-55F9-AA45-62A06C2BB501

Figure 3A

####### Material examined.

1 ♂, 2 ♀♀; St. 7; 29°22.497'N, 47°45.183'E; 02 Feb. 2014; 1 ♀, 6 praniza larvae; St. 8; 29°23.047'N, 47°50.192'E; 3 Feb. 2014; 1 ♀, 6 praniza larvae; St. 10; 29°21.401'N, 47°56.390'E; 22 Feb. 2014; 1 ♂; St. 11; 29°21.631'N, 47°57.204'E; 9 Dec. 2013; 1 ♀; St. 12; 29°23.481'N, 47°59.800'E; 08 Dec. 2013; 3 ♀♀, 8 praniza larvae; St. 19; 29°04.431'N, 48°08.676'E; 10 Dec. 2014; 2 ♀♀, 1 praniza larva; St. 21; 29°00.071'N, 48°09.853'E; 16 Feb 2014; 1 ♀, 3 praniza larvae; St. 25; 28°38.813'N, 48°23.429'E; 3 Mar. 2014; 3 ♀♀, 1 ♂, 50 praniza larvae; St. 26; 28°34.794'N, 48°24.078'E; 4 Mar. 2014; 5 ♂♂, 9 ♀♀, 6 praniza larvae; St. 27; 28°40.778'N, 48°39.207'E; 11 Nov. 2014; 1 ♂, 3 praniza larvae; St. 28; 28°40.939'N, 48°39.196'E; 11Nov.2014; 8 ♂♂, 16 juveniles, 50 ♀♀, 3 praniza larvae; St. 30; 28°49.105'N, 48°46.553'E; 10 Nov. 2014; 3 ♀♀, 6 juveniles; St. 31; 28°49.022'N, 48°46.607'E; 10 Nov. 2014; 50 ♀♀ and praniza larvae; St. 32; 29°04.278'N, 48°29.655'E; 9 Nov. 2014; 3 ♀♀; St. 35; 29°23.710'N, 48°24.136'E; 25 Dec. 2014; 4 ♂♂, 2 sub adults ♂♂, 3 praniza larvae; St. 36; 29°23.629'N, 48°23.958'E; 24 Dec. 2014; 4 praniza larvae; St. 38; 29°28.049'N, 48°17.838'E; 22 Dec. 2014.

####### Remarks.

The specimen is closely related to *Gnathialuxata* Kensley, Schotte & Poore, 2009 from Khawr Musharraba, Saudi Arabia, Persian Gulf. However, it differs from *G.luxata* by having a larger and conical mediofrontal process and bifid superior frontolateral process instead of a conical process. Also, the supraocular lobe is blunt and oblique rather than simply rounded.

**Figure 3.**
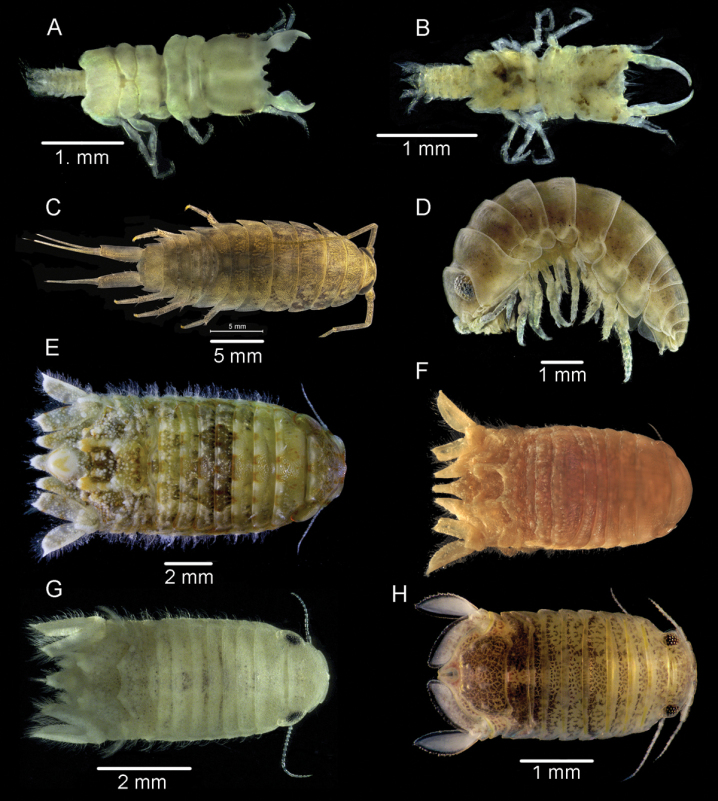
**A***Gnathia* sp. from Al-Nuwaiseeb **B***Elaphognathia* sp., from Al-Shamaimah **C***Ligiapersica* from Al-Nuwaiseeb **D***Tylosmaindroni* Giordani Soika, 1954 from Kubbar Island **E***Cymodocedelvarii* Khalaji-Pirbalouty, Bruce & Wägele, 2013 from Al-Nuwaiseeb **F***C.fuscina* Schotte & Kensley, 2005 from USNM **G***C.waegelei* Khalaji-Pirbalouty & Raupach,, 2014 from Al-Nuwaseeb **H***Dynamenellagranulata* Javed & Ahmed, 1988 from Um-Almaradim.

####### Distribution.

New record for Kuwait.

##### Genus *Elaphognathia* Monod, 1926

###### ﻿ Elaphognathia

Taxon classificationAnimaliaIsopodaGnathiidae

sp.

251C742C-9BF2-5015-A9AE-21787F90D6B3

Figure 3B

####### Material examined.

1 ♂; St. 3; 24°11.2014'N, 48°07.850'E; 24 Nov. 2014.

####### Remarks.

The specimen is similar to *E.gladia* Kensley, Schotte & Poore, 2009 in having the long, thin saber-like mandible from Somalia. However, it differs from *E.gladia* in having a mandible with only one conical lobe at its base rather than two and having an acute mediofrontal process (vs. absent in *E.gladia*).

####### Distribution.

New record for Kuwait.

#### Family Bopyridae Rafinesque, 1815

##### Genus *Epipenaeon* Nobili, 1906

###### ﻿ Epipenaeon elegans

Taxon classificationAnimaliaIsopodaBopyridae

Chopra, 1923

888CFCE7-4395-5342-BDA3-5F26B0A3FA2E

Epipenaeon elegans Chopra, 1923: 454–456, figs 6–11, Ganges Delta, India (type locality); Dawson, 1958: 240; Tareen, 1982: 159–160; Abu-Hakima 1984: 51–58; Mathews et al. 1988: 53–62; Eslami & Mokhayer, 2002: 89–95; An et al. 2015: 2033.

####### Distribution.

India; Kuwait; Abu Ali and Tarut (Saudi Arabia); Boushehr port (Iran).

##### Genus *Parabopyrella* Markham, 1985

###### ﻿ Parabopyrella

Taxon classificationAnimaliaIsopodaBopyridae

sp.

94B883AF-CCC5-5770-8B46-314535D72329

####### Material examined.

1 ♂, 1 ♀; St.8; 29°23.047'N, 47°50.192'E; 3 Feb. 2014.

####### Remarks.

Parasite, found on the gill of the common alpheid shrimp in Kuwait the *Alpheuslobidens* De Haan, 1849.

####### Distribution.

New record for Kuwait.

### ﻿Suborder Oniscidea Latreille, 1802

**Family Ligiidae Brandt & Ratzeburg, 1831**

#### Genus *Ligia* Fabricius, 1798

##### ﻿ Ligia persica

Taxon classificationAnimaliaIsopodaLigiidae

Khalaji-Pirbalouty & Wägele, 2010

36ED24AF-1E34-5741-BE18-9E5F4A6F18E9

Figure 3C

Ligia persica Khalaji-Pirbalouty & Wägele, 2010b: 136–149, figs 2–7; Kish Island, Iran (type locality).
Ligia exotica Roux, 1828. – Jones, 1986: 148, pl. 40.

###### Material examined.

1 ♀; St. 7; 29°22.497'N, 47°45.183'E; 2 Feb. 2014; 4 ♂♂, 2 ♀♀; St. 8; 29°23.047'N, 47°50.192'E; 3 Feb. 2014; 3 ♂♂, 17 ♀♀; St. 12; 29°23.481'N, 47°59.800'E; 8 Dec. 2013 20 ♂♂ and ♀♀; St. 13; 29°21.979'N, 48°01.344'E; 19 Jan. 2014; 4 ♂♂, 6 ♀♀; St. 26; 28°34.794'N, 48°24.078'E; 4 Marc. 2014; 4 ♂♂, 4 ♀♀; St. 28; 28°40.939'N, 48°39.196'E; 11 Nov. 2014.

###### Distribution.

Iran, Oman, and United Arab Emirates (Taiti and Checcucci 2011; Khalaji-Pirbalouty and Wägele 2010), new record for Kuwait.

####### Family Olibrinidae Budde-Lund, 1913

#### Genus *Olibrinus* Budde-Lund, 1913

##### ﻿ Olibrinus antennatus

Taxon classificationAnimaliaIsopodaOlibrinidae

Budde-Lund, 1902

CEC8FE68-199B-5DFA-8EC8-5054BDA01561

Olibrinus antennatus Budde-Lund, 1902: 379, Malaysia (type locality); Schmalfuss, 2003: 182; Taiti & Ferrara, 2004: 223, pl. 4.

###### Material examined.

1 ♀; St. 3; 29°39.403'N, 48°07.450'E; 24 Nov. 2014.

###### Distribution.

Indian Ocean (Taiti and Ferrara 2004), coastal waters of Iran (Khalaji-Pirbalouty, unpublished data), new record for Kuwait.

####### Family Tylidae Milne-Edwards, 1840

#### Genus *Tylos* Audouin, 1826

##### ﻿ Tylos maindroni

Taxon classificationAnimaliaIsopodaTylidae

Giordani Soika, 1954

2B8EF8DB-1845-56F5-95A5-744FDBE7D46B

Figure 3D

Tylos maindroni Giordani Soika, 1954: 76, figs 8, 9, pl. 10, Oman Sea, Muscat (type locality); Ferrara & Taiti, 1986: 94; Taiti & Ferrara, 1991: 213, fig. 3; Taiti et al. 2000: 148.
Tylos sp. Jones, 1986: 149, pl. 40.

###### Material examined.

2 juveniles; St. 4; (1 ♀); St. 28; 11 Nov. 2014; 2 ♂♂, 2 ♀♀; St. 33; 29°04.377'N, 48°29.472'E; (1 ♀, 3 juveniles); St. 35; 29°23.710'N, 48°24.136'E; 25 Dec. 2014; 1 ♀, 4 juveniles; St. 38; 29°22.726'N, 48°26.269'E; 22 Dec. 2014.

###### Distribution.

Oman, Kuwait (Taiti and Ferrara 1991); Bandar-e-Charak, Bandar-e Bostanoo, Iran (Khalaji-Pirbalouty, unpublished data).

### ﻿Suborder Sphaeromatidea Wägele, 1989

**Family Sphaeromatidae Latreille, 1825**

#### Genus *Cymodoce* Leach, 1814

##### ﻿ Cymodoce delvarii

Taxon classificationAnimaliaIsopodaSphaeromatidae

Khalaji-Pirbalouty, Bruce & Wägele, 2013

DBA64C0D-E100-5445-8D34-02E985AEE844

Figure 3E

Cymodoce delvarii Khalaji-Pirbalouty et al.,, 2013: 523–528, figs 16–19; Boushehr Province, Iran (type locality).
Cymodoce richardsoniae Jones, 1986: 149, pl. 40 [not C.richardsoniae Nobili, 1906; misidentification].

###### Material examined.

1 ♂, 1 ♀, 1 subadult ♂, 1 juvenile; St. 3; 29°39.403'N, 48°07.850'E; 24 Nov. 2014; 6 ♂♂ 25 ♀♀, 6 sub adult ♂♂; St. 12; 29°23.481'N, 47°59.800'E; 8 Dec. 2013; 1 juvenile St.15; 29°16.496'N, 48°05.407'E; 18 Dec. 2013; 1 ♀, 1 sub-adult ♂, 1 juvenile; St.18; 29°06.041'N, 48°08.323'E; 1 Feb. 2014; 1 ♀; St.19; 29°04.431'N, 48°08.676'E; 10 Dec. 2014; 2 juveniles; St. 25; 28°38.813'N, 48°23.429'E; 3 Mar. 2014; 4 ♀♀, many juveniles; St. 26; 28°34.794'N, 48°24.078'E; 4 Mar. 2014; 1 ♀; St. 28; 28°40.939'N, 48°39.196'E; 11 Nov. 2014; 1 ♀; St. 32; 29°04.278'N, 48°29.655'E; 9 Nov. 2014; 1 ♂, 15 ♀♀; St. 34; 29°22.726'N, 48°26.269'E; 10 Feb. 2016; 1 ♀; St. 35; 29°23.710'N, 48°24.136'E; 25 Dec. 2014; 2 ♂♂, 26 ♀♀, many juveniles; St. 36; 29°23.629'N, 48°23.958'E; 24 Dec. 2014.

###### Distribution.

Bousher Province, Iran (Khalaji-Pirbalouty, Bruce and Wägele 2013), new record for Kuwait.

##### ﻿ Cymodoce fuscina

Taxon classificationAnimaliaIsopodaSphaeromatidae

Schotte & Kensley, 2005

55D33660-9034-5770-8A22-2C93AF2D382E

Figure 3F

Cymodoce fuscina Schotte & Kensley, 2005: 1245–1248, figs 19–20, Safaniya and Manifa, Saudi Arabia (type locality); Ulman et al. 2017: 27.
Cymodoce sp. Jones, 1986: 149, pl. 40.

###### Material examined.

2 ♂♂ and 3 ♀♀; Kuwait Fishery Station (from Smithsonian Natural History Museum collection, USNM 1145230).

###### Distribution.

Saudi Arabia, United Arab Emirates, the Mediterranean basin, Greece (Schotte and Kensley 2005; Ulman et al. 2017), new record for Kuwait.

##### ﻿ Cymodoce waegelei

Taxon classificationAnimaliaIsopodaSphaeromatidae

Khalaji-Pirbalouty & Raupach, 2014

8DD9F7F1-B0BE-5722-BBAC-E38D943EA7B5

Figure 3G

Cymodoce waegelei Khalaji-Pirbalouty & Raupach, 2014: 242–249, figs 7–12, Boushehr Province, Iran (type locality); Khalaji-Pirbalouty et al. 2015: 34, fig. 2.

###### Material examined.

2 ♂♂, 3 ♀♀; St. 25; 28°38.813'N, 48°23.429'E; 3 Mar. 2014; 4 ♂♂, 9 ♀♀; St. 26; 28°34.794'N, 48°24.078'E; 4 Mar. 2014; 1 ♂, 1 ♀ St. 27; 28°40.778'N, 48°39.207'E; 11 Nov. 2014.

###### Distribution.

Bousher Province and Hengam Island, Iran (Khalaji-Pirbalouty and Raupach 2014; Khalaji-Pirbalouty et al. 2015), new record for Kuwait.

#### Genus *Dynamenella* Hansen, 1905

##### ﻿ Dynamenella granulata

Taxon classificationAnimaliaIsopodaSphaeromatidae

Javed & Ahmed, 1988

48B0A4BE-9C21-5DFB-B0B9-8B2B1092CA88

Figure 3H

Dynamenella granulata Javed & Ahmed, 1988: 234–236, figs 1–3, Karachi coast, Pakistan (type locality).

###### Materials examined.

1 juvenile; St. 25; 28°38.813'N, 48°23.429'E; 3 Mar. 2014; 1 sub-adult ♂, 2 ♀♀, 1 juvenile; St. 28; 28°40.939'N, 48°39.196'E; 11 Nov. 2014; 4 sub-adults ♂♂, 5 ♀♀, 5 juveniles; St. 33; 29°04.377'N, 48°29.472'E; 9 Nov. 2014.

###### Distribution.

Pakistan and Iran coasts (Javed & Ahmed, 1988; Khalaji-Pirbalouty unpublished data), new record for Kuwait.

#### Genus *Heterodina* Schotte & Kensley, 2005

##### ﻿ Heterodina mccaini

Taxon classificationAnimaliaIsopodaSphaeromatidae

Schotte & Kensley, 2005

1B083E25-E086-5B70-8594-2ACCD1A7A653

Figure 4A

Heterodina mccaini Schotte & Kensley, 2005: 1259–1261, figs 27, 28, Manifa, Saudi Arabia (type locality).

###### Material examined.

> 100 ♂♂ and ♀♀; St. 7; 29°22.497'N, 47°45.183'E; 2 Feb. 2014; 1 ♀; St. 8; 29°23.047'N, 47°50.192'E; 7 ♀♀; St. 12; 29°23.481'N, 47°59.800'E; 8 Dec. 2013; 8 ♂♂, > 100 ♀♀ and Juveniles; St. 19; 47°59.800'N, 48°08.676'E; 10 Dec. 2014; 1 ♀; St. 21; 29°00.071'N, 48°09.853'E; 16 Feb. 2014; 5 ♂♂, 23 ♀♀; St. 25; 28°38.813'N, 48°23.429'E; 3 Mar. 2014; > 100 ♂♂ and ♀♀; St. 26; 28°34.794'N, 48°24.078'E; 4 Mar. 2014; 2 ♀♀, 1 juvenile; St. 32; 29°04.278'N, 48°29.655'E; 9 Nov. 2014; 2 ♀♀; St. 34; 29°22.726'N, 48°26.269'E; 10 Feb. 2016; 1 ♂, 1 ♀; St. 37; 29°25.625'N, 48°20.307'E; 23 Dec. 2014; 3 ♀♀; St. 38; 29°28.049'N, 48°17.838'E; 22 Dec. 2014.

###### Distribution.

Manifa and Ras Tanajib, Saudi Arabia (Schotte and Kensley 2005), new record for Kuwait.

**Figure 4.**
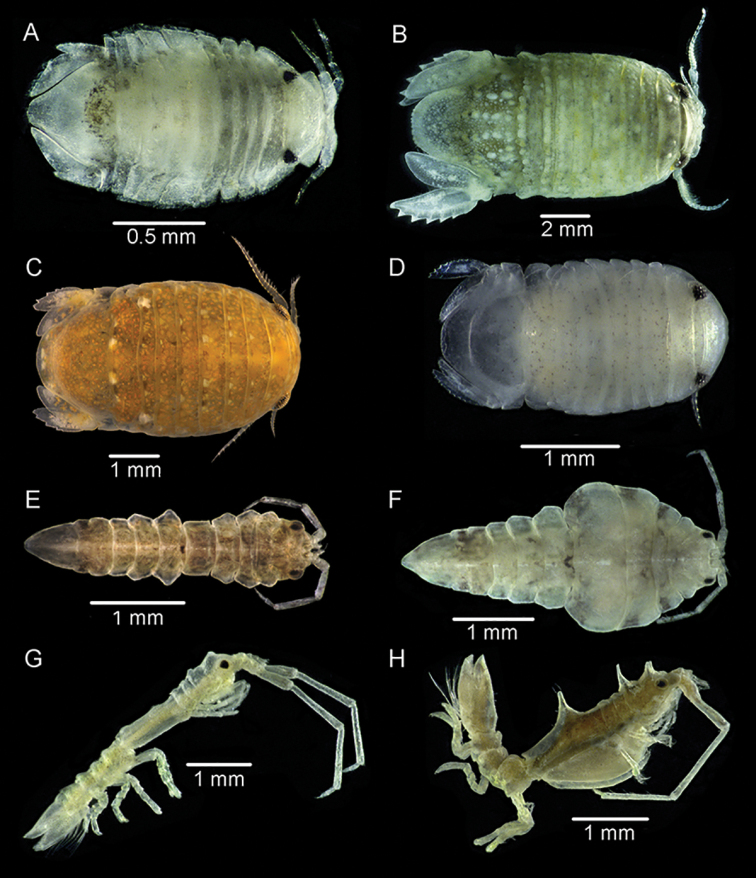
**A***Heterodinamccaini* Schotte & Kensley, 2005 from Al-Nuwaiseeb **B***Sphaeromawalker* Stebbing, 1905 from Al-Zhor **C***S.khalijfarsi* Khalaji-Pirbalouty & Wägele, 2010 from Boubyan Island **D***Sphaeromopsissarii* Khalaji-Pirbalouty & Wägele, 2009 from Kubbar Island **E***Arcturinoidesangulata* Kensley, Schotte & Poore, 2007 ♂, from Al-Doha **F***A.angulata* ♀ from Al-Doha **G***Astacillamccaini* Kensley, Schotte & Poore, 2007 ♂, from Failaka Island **H***A.mccaini* ♀, from Failaka Island.

#### Genus *Sphaeroma* Bosc, 1802

##### ﻿ Sphaeroma walkeri

Taxon classificationAnimaliaIsopodaSphaeromatidae

Stebbing, 1905

1ADA4099-E967-5A0A-BFDD-345FEBBEE3A2

Figure 4B

Sphaeroma walkeri Stebbing, 1905: 31–33, pl. VII, Jokkenpiddi Paar, Sri Lanka (type locality). Latest synonymies to the species can be found in Martínez-Laiz et al., (2018: 13).

###### Material examined.

8 ♂♂, 5 ♀♀, 10 juveniles; St. 24; 28°44.502'N, 48°22.950'E; 8 Jan. 2015; 9 ♀♀; St. 25; 28°38.813'N, 48°23.429'E; 3 Mar. 2014.

###### Distribution.

*Sphaeromawalkeri* is one the most widespread species of the marine isopods, reported along the Indian, Atlantic, and Pacific oceans coastal zones (Khalaji-Pirbalouty and Wägele 2010c; Martínez-Laiz et al. 2018).

##### ﻿ Sphaeroma khalijfarsi

Taxon classificationAnimaliaIsopodaSphaeromatidae

Khalaji-Pirbalouty & Wägele, 2010

27441AEF-1DE5-5C61-B4B2-F39E5B5CDE28

Figure 4C

Sphaeroma khalijfarsi Khalaji-Pirbalouty & Wägele, 2010c: 3–9, figs 1–5, Qeshm Island, Iran (type locality).

###### Material examined.

3 ♀♀, 25 juveniles; St. 4; 29°34.849'N, 48°10.248'E; 25 Nov. 2014; 1 juvenile; St. 17; 29°08.154'N, 48°07.985'E; 4 Jan. 2014; 2 ♀♀; St. 26; 28°34.794'N, 48°24.078'E; 4 Mar. 2014; 2 ♂♂, 6 ♀♀, 6 juveniles; St. 39; 29°38.993'N, 48°18.830'E; 24 Jan. 2015; 4 ♂♂, 25 ♀♀, 21 juveniles; St. 40; 29°48.093'N, 48°21.975'E; 25 Jan. 2015.

###### Distribution.

Qeshm Island, Bandare Abbas, Bandare Kolahi, Iran (Khalaji-Pirbalouty and Wägele 2010c), new record for Kuwait.

##### ﻿ Sphaeroma annandalei

Taxon classificationAnimaliaIsopodaSphaeromatidae

Stebbing, 1911

AE51027D-796A-53C8-9555-BD3459ED4948

Sphaeroma annandalei Stebbing, 1911: 181, pl. X, West Bengal, India (type locality); Barnard, 1936: 174; Barnard, 1940: 405; Pillai, 1955: 134, figs 23–35, pl. VII; Joshi & Bal, 1959: 62; Kensley, 1978: 113; Jones, 1986: 149, pl. 40; Khalaji-Pirbalouty & Wägele, 2010: 31–37, figs 1–5.
Sphaeroma irakiensis irakiensis Ahmed, 1971: 77–79, fig. 1.

###### Distribution.

India, Habbanyyah Lake, and Shat Al- Arab River (Iraq); Arvand Kenar (Iran); Kuwait.

#### Genus *Sphaeromopsis* Holdich & Jones, 1973

##### ﻿ Sphaeromopsis sarii

Taxon classificationAnimaliaIsopodaSphaeromatidae

Khalaji-Pirbalouty & Wägele, 2009

C99F521E-F560-5F6C-944A-2B3CB12CF4DC

Figure 4D

Sphaeromopsis sarii Khalaji-Pirbalouty & Wägele, 2009: 34–42, figs 1–5, Kish Island, Iran (type locality).

###### Material examined.

1 ♀; St. 4; 29°34.849'N, 48°10.248'E l; 25 Nov. 2014; 3 ♀♀l; St. 12; 29°23.481'N, 47°59.800'E; 8 Dec. 2013; 100 ♂♂ and ♀♀; St. 15; 29°16.496'N, 48°05.407'E; 2 ♂♂, 15 ♀♀; St. 18; 29°06.041'N, 48°08.323'E; 1 Feb. 2014; 1 ♂, 10 ♀♀; St. 21; 29°00.071'N, 48°09.853'E; 16 Feb. 2014; 2 ♂♂, 12 ♀♀, 2 Juveniles; St. 24; 28°44.502'N, 48°22.950'E; 8 Jan. 2015; 3 ♂♂, 8 ♀♀; St. 25; 28°38.813'N, 48°23.429'E; 3 Mar. 2014; 1 ♀; St. 26; 28°34.794'N, 48°24.078'E; 4 Mar. 2014; 22 ♂♂, 9 ♀♀, 2 juveniles; St. 27; 28°40.778'N, 48°39.207'E; 11 Nov. 2014; 28 ♂♂, 31 ♀; St. 28; 28°40.939'N, 48°39.196'E; 11 Nov, 2014; 35 ♂♂ and ♀♀; St. 29; 28°40.960'N, 48°39.173'E; 11 Nov. 2014; > 100 ♂♂ and ♀♀; St. 30; 28°49.105'N, 48°46.553'E; 10 Nov. 2014; 3 ♂♂, 43 ♀♀; St. 31; 28°49.022'N, 48°46.607'E; 10 Nov. 2014; > 100 ♂♂ and ♀♀; St. 32; 29°04.278'N, 48°29.655'E; 9 Nov. 2014; 9 ♂♂, 12 ♀♀, 3 juveniles; St. 33; 29°04.377'N, 48°29.472'E; 9 Nov. 2014 13 ♂♂, 14 ♀♀; St. 34; 29°22.726'N, 48°26.269'E; 10 Feb. 2016; > 100 ♂♂ and ♀♀, St. 36; 29°23.629'N, 48°23.958'E; 10 Feb. 2016; > 100 ♂♂ and ♀♀; St. 37; 29°25.625'N, 48°20.307'E; 23 Dec. 2014.

###### Distribution.

Kish, Qeshm, Hengam Islands, Iran (Khalaji-Pirbalouty and Wägele 2009; Khalaji- Pirbalouty et al. 2015), new record for Kuwait.

### ﻿Suborder Valvifera Sars, 1882

#### Family Arcturidae Sars, 1897

##### Genus *Arcturinoides* Kensley, 1977

###### ﻿ Arcturinoides angulata

Taxon classificationAnimaliaIsopodaArcturidae

Kensley, Schotte & Poore, 2007 Figures 4E, 7F

11F249DD-7105-5B83-B945-BA0F3C648AD5

Arcturinoides angulata Kensley et al., 2007: 433–436, figs 3, 4, Kuwait Bay (type locality).

####### Material examined.

1 ♀; St. 7; 29°22.497'N, 47°45.183'E; 2 Feb. 2014; 1 ♀; St. 8; 29°23.047'N, 47°50.192'E; 3 Feb. 2014; 2 ♂♂; St. 34; 29°22.726'N; 48°26.269'E; 10 Feb. 2016; 1 ♂; St. 35; 29°23.710'N, 48°24.136'E; 25 Dec. 2014.

####### Distribution.

United Arab Emirates, Kuwait Bay, Kuwait (Kensley et al. 2007).

##### Genus *Astacilla* Cordiner, 1793

###### ﻿ Astacilla mccaini

Taxon classificationAnimaliaIsopodaArcturidae

Kensley, Schotte & Poore, 2007

71B9B655-C38B-5842-93D7-D2DC3F04315E

Figure 4G, H

Astacilla mccaini Kensley et al., 2007: 437–440, figs 5, 6, Manifa Bay, Saudi Arabia (type locality).

####### Material examined.

10 ♂♂; St. 20; 29°8.043'N, 48°9.139'E; 28 Sep. 2014; 1 ♀; St. 21; 29°00.071'N, 48°09.853'E; 16 Feb. 2014; 1 ♂; St. 34; 29°22.726'N, 48°26.269"E; 10 Feb. 2016; 1 ♂; St. 35; 29°23.710'N, 48°24.136'E; 25 Dec. 2014; 6 ♂♂, 2 ovigerous ♀♀, 2 juveniles; St. 36; 29°23.629'N, 48°23.958'E; 25 Dec. 2014.

####### Distribution.

Manifa Bay, Saudi Arabia; Kuwait Bay, Kuwait (Kensley et al. 2007).

### ﻿Suborder Asellota Latreille, 1802

**Family Paramunnidae Vanhöffen, 1914**

#### *Heterosignum* Gamô, 1976

**Type species.**

*Heterosignummutsuensis* Gamô, 1976

##### ﻿ Heterosignum

Taxon classificationAnimaliaIsopodaParamunnidae

sp.

352BF6D4-077A-589A-AA7C-4781C592FD11

###### Material examined.

3 ♀; St. 28; 28°40.939'N, 48°39.196'E; 11 Nov, 2014; 1 ♀; St. 25; 28°38.813'N, 48°23.429'E; 3 Mar. 2014.

###### Distribution.

New record for Kuwait.

## ﻿Discussion

Bowmen and Tareen (1983) were the first to study Kuwait’s isopod fauna, recording nine species of Cymothoidae, all ectoparasitic on marine fishes (Table 6). Jones (1986) included six isopod species in his ‘Field Guide to the Seashores of Kuwait’: *Apanthurasandalensis*, *Ligiaexotica*, and *Cymodocerichardsoniae* are reidentified as *Amakusanthura* sp., *L.persica*, and *C.delvarii*, respectively. Moreover, *Cymodoce* sp. of Jones (1986) is reidentified as *Cymodocefuscina* and *Tylos* sp. is identified as *Tylosmaindroni*. The widespread supratidal isopod *Tylosmaindroni* was previously reported from Kuwait by Taiti and Ferrara (1991). However, *Sphaeromaannandalei* Stebbing, 1911 was not found in the current study: the known distribution of *S.annandalei* is from the West Bengal estuaries in India to the Arvandroud (Shatt-Al-Arab) riverbanks between Iran and Iraq (Khalaji-Pirbalouty and Wägele 2010).

Two additional species, *Arcturinoidesangulata* and *Astacillamccaini*, were collected from Kuwait Bay by Kensley et al. (2007). In monitoring the fauna of recently dredged canals in the Al-Khiran area of Kuwait, Jones and Nithyanandan (2012) discovered and described two new isopod species from Kuwait and mentioned the occurrence of a third, increasing the valid species of Isopoda recorded from Kuwait to 17. With the present survey, we now count 38 species of Isopoda, more than doubling Kuwait’s known isopod fauna. Twenty-one of the 25 species collected for this study represent first records for Kuwait (Table 2). Only four of these 25 species were reported previously: *Eurydiceperaticis*, *Tylosmaindroni*, *Arcturinoidesangulata*, and *Astacillamccaini*.

The geographical distribution of isopod species in Kuwait waters show very different patterns. The burrowing isopod *Sphaeromawalkeri* was found living in soft rocks in the high intertidal area of the Al-Zour coast. The type locality of this species is Sri Lanka, and it has been considered restricted to the northern Indian Ocean. This thermophilic species is also tolerant to a range of salinities, and its distribution is worldwide in the tropics (Ríos-Touma et al. 2017). The ranges of other species are also limited to the Indian Ocean. For example, *Lanociragardineri*, is widely distributed from western Australia (Bruce and Sidabalok 2011) and the Maldives, Kenya, and Madagascar (Delaney 1989). Delaney (1989) recorded it from the Khor Abdullah estuary, Iraq, in the northwestern Gulf. Tolerance of salinity fluctuations is believed to be a primary reason for the wide distribution of this species throughout the Indian Ocean. Other species, such as *Dynamenellagranulata*, and *Atarbolanaexoconta*, are widely distributed along the northeastern coast of the Gulf and along the Pakistani coast. Their distribution pattern is similar to some brachyuran decapods as suggested by Apel and Türkay (1999) and Naderloo et al. (2011). According to this distribution pattern, the fiddler crab fauna of the southern and western Gulf is similar in East Africa, the Gulf of Aden, and the Red Sea. At the same time, the fauna of the northeastern parts of the Persian Gulf is also somewhat similar to that of the northeastern Arabian Sea coasts of Pakistan and India. Finally, some of the known species of isopoda (e.g., *Heterodinamccaini* ; *Sphaeromakhalijfarsi*, and *Sphaeromopsissarii*) are indigenous to the Gulf.

The new results reveal a low species richness of Isopoda in Kuwait waters compared to the adjacent regions of the Indian Ocean. Based on Kensley’s (2001) isopod checklist, the Indian Ocean exhibits a high species diversity of more than 1000 species. Of these, 268 species inhabit the Indian coastal region, and fewer than half that number, 121 species, has been recorded from Pakistan’s coast by Kazmi et al. (2002). The apparently low species richness of Isopoda in the Kuwait region compared to that of other areas of the Indian Ocean is due to Kuwait’s limited coastline, less than 200 km, but also to the Gulf’s young age, less than 6,000 years BP (Sheppard et al. 2010), and the harsh environmental conditions. The age of the environment is an essential factor for the evolution of diversity (Gaston and Chown 1999). The seabed regions of the Gulf presently at depths of 4–6 m have only been submerged for 3,000–4,000 years (Sheppard et al. 2010). Therefore, the current coastal habitat development is comparatively young.

The harsh environmental conditions in Kuwait coastal zone arise from high temperatures and high salinity. Salinities exceed 40 PSU, and summer temperatures often exceed 35 °C. For instance, from 2000 to 2013, the mean seawater temperature in Kuwait Bay was 23.6 °C with a range of 9.7–36.0 °C, and salinity ranged from 30–46 PSU (Al-Yamani et al. 2004). Furthermore, extreme air temperatures with highs up to 55°C in the summer months and winter lows around freezing are known from Kuwait (Jones 1986).

However, a comparison between this study and restricted localities of similar size suggested no lower diversity in Kuwait. Brusca (1987) reported 36 species of marine isopods from the Galapagos. Seventeen species of these were shallow-water species from the littoral to a depth of 100 m. Furthermore, Kensley (1984) identified only 24 species of isopods from the Belizean reef crest. The low species composition of these studies may arise from limited sampling. This study focuses on the Kuwaiti shoreline; therefore, many species living in sub-tidal depths were not collected.

Some isopod species appear to be introduced into Kuwait Bay from outside of the Gulf. For example, *Cymodocefuscina* and *C.waegelei* were found in the subtidal zone of the Iranian and Arabian coasts of the Gulf, but were also recently reported from the Mediterranean basin, Greece (Ulman et al. 2017) and Egypt (pers. obs.). This distribution supports the hypothesis of a human- assisted introduction, such as through ballast water discharge. According to the Public Relations Department of Hormozgan Ports, Iran, ca. 53,000 tanker and cargo ships enter the Gulf annually and ca. 40% of the world’s total oil transportation passes through the Strait of Hormuz (Al-Yamani et al. 2015). In this context, ships transport a billion tonnes of ballast water annually, so although this intertidal study was comprehensive, it was only limited to sampling in the intertidal zone. Repeated sampling during different seasons as well as subtidal investigations would certainly increase Kuwait’s known isopod fauna.

The present study provides a baseline account of Kuwait’s coastal zone isopod fauna. The next step will be evaluating their ecology and conservation status. As Kuwait is one of the major oil exporters, invasive species are a significant issue, mainly due to the discharge of ballast water from oil tankers and cargo ships. Therefore, prevention is crucial for decision-making and implementation of invasion control and detection of new exotics. The results of this study highlight the need for further morphological as well as molecular studies to clarify the taxonomic status of some specimens, and a larger sampling effort in deeper waters of this area.

## Supplementary Material

XML Treatment for Amakusanthura

XML Treatment for Eisothistos

XML Treatment for Atarbolana exoconta

XML Treatment for Baharilana kiabii

XML Treatment for Cirolana tarahomii

XML Treatment for Eurydice arabica

XML Treatment for Eurydice marzouqui

XML Treatment for Eurydice peraticis

XML Treatment for Metacirolana

XML Treatment for Lanocira gardineri

XML Treatment for Anilocra monoma

XML Treatment for Catoessa gruneri

XML Treatment for Joryma sawayah

XML Treatment for Nerocila arres

XML Treatment for Nerocila sigani

XML Treatment for Nerocila phaiopleura

XML Treatment for Mothocya

XML Treatment for Gnathia

XML Treatment for Elaphognathia

XML Treatment for Epipenaeon elegans

XML Treatment for Parabopyrella

XML Treatment for Ligia persica

XML Treatment for Olibrinus antennatus

XML Treatment for Tylos maindroni

XML Treatment for Cymodoce delvarii

XML Treatment for Cymodoce fuscina

XML Treatment for Cymodoce waegelei

XML Treatment for Dynamenella granulata

XML Treatment for Heterodina mccaini

XML Treatment for Sphaeroma walkeri

XML Treatment for Sphaeroma khalijfarsi

XML Treatment for Sphaeroma annandalei

XML Treatment for Sphaeromopsis sarii

XML Treatment for Arcturinoides angulata

XML Treatment for Astacilla mccaini

XML Treatment for Heterosignum
